# A new macrolepidopteran moth (Insecta, Lepidoptera, Geometridae) in Miocene Dominican amber

**DOI:** 10.3897/zookeys.965.54461

**Published:** 2020-09-03

**Authors:** Weiting Zhang, Chungkun Shih, YuHong Shih, Dong Ren

**Affiliations:** 1 Hebei GEO University, 136 Huaiandonglu, Shijiazhuang 050031, China Hebei GEO University Shijiazhuang China; 2 State Key Laboratory of Palaeobiology and Stratigraphy, Nanjing Institute of Geology and Palaeontology, CAS, Nanjing 210008, China Nanjing Institute of Geology and Palaeontology Nanjing China; 3 College of Life Sciences and Academy for Multidisciplinary Studies, Capital Normal University, 105 Xisanhuanbeilu, Haidian District, Beijing 100048, China Capital Normal University Beijing China; 4 Department of Paleobiology, National Museum of Natural History, Smithsonian Institution, Washington, DC 20013-7012, USA National Museum of Natural History Washington United States of America; 5 Laboratorio Dominicano De Ambar Y Gemas, Santo Domingo, Dominican Republic Laboratorio Dominicano De Ambar Y Gemas Santo Domingo Dominican Republic

**Keywords:** *
chunjenshihi*, Ennominae, extinct, fossil, taxonomy

## Abstract

A new genus and species of fossil moth, *Miogeometrida chunjenshihi* Zhang, Shih & Shih, **gen. et sp. nov.**, assigned to Geometridae, is described from Miocene Dominican amber dating from 15–20 Mya. The new genus is characterized by the forewing without a fovea, R_1_ not anastomosing with Sc, no areole formed by veins R_1_ and Rs, R_1_ and Rs_1_ completely coincident, M_2_ arising midway between M_1_ and M_3_, anal veins 1A and 2A fused for their entire lengths; and the hind wing with Rs running close to Sc + R_1_ and M_2_ absent.

## Introduction

Geometridae, the second most species-rich family of Lepidoptera, comprise approximately 24,000 described species (van Nieukerken et al. 2011; Murillo-Ramos et al. 2019). Geometridae are macrolepidopterans characterized by the presence of unique tympanal organs at the base of the abdomen, and the prolegs of their larvae reduced to two pairs causing the larvae to move by ‘looping’ (Minet and Scoble 1999). Geometridae were once classified into six subfamilies: Geometrinae, Ennominae, Sterrhinae, Larentiinae, Archiearinae and Oenochrominae*sensu lato* (Holloway 1994, 1996, 1997; Minet and Scoble 1999), but this classification was not fully satisfactory due to the fact that Oenochrominae*sensu lato* are a polyphyletic group (Scoble and Edwards 1990). Oenochrominae*sensu lato* were further divided into Oenochrominae*sensu stricto*, Desmobathrinae, Orthostixinae, and Alsophilinae, forming a classification system of nine subfamilies (Yamamoto and Sota 2007), but later Alsophilinae was subsumed in Ennominae (Wahlberg et al. 2010). Sihvonen et al. (2011) provided a comprehensive phylogeny of the Geometridae, and they found the previously recognized subfamilies to be monophyletic except the Oenochrominae + Desmobathrinae complex, which is a polyphyletic assemblage of taxa, and the Orthostixinae, which was positioned within the Ennominae. The systematic status of Orthostixinae remains uncertain, although Orthostixinae were synonymized with Desmobathrinae by Beljaev (2016). Systematic updates and annotated checklists of Western Palaearctic Geometridae were provided in "The Geometrid Moths of Europe" series (Hausmann 2001, 2004; Mironov 2003; Hausmann and Viidalepp 2012; Skou and Sihvonen 2015; Müller et al. 2019). Murillo-Ramos et al. (2019) established a new subfamily Epidesmiinae and transferred eight genera from Oenochrominae*sensu stricto* to Epidesmiinae.

The age of Geometroidea was calculated to trace back to 83 Mya (Wahlberg et al. 2013), and the age of Geometridae was estimated at ca 54 Mya (62–48 Mya, Yamamoto and Sota 2007). Recently, Kawahara et al. (2019) inferred a comprehensive phylogeny of Lepidoptera, and they dated the oldest members of the Lepidoptera crown group in the Late Carboniferous (ca 300 Mya), and speculated the ancestors of Geometroidea appeared in the Late Cretaceous. To date, 18 fossil records of Geometridae have been formally reported (Table 1). Harris and Raine (2002) reported a Late Cretaceous (Albian-Turonian, 113–89.8 Mya) lepidopterous genitalic fragment from New Zealand, and deemed its affinity probably lies within Geometridae, but Sohn et al. (2012) regarded the available characters insufficient to support a family-level diagnosis. The Eocene (56–33.9 Mya) species, *Eogeometer vadens* Fischer, Michalski & Hausmann, 2019, *Geometridites larentiiformis* Jarzembowski, 1980 and Hydriomena ? protrita Cockerell, 1922, respectively from the Baltic, UK, and USA, are believed to be the earliest representatives of Geometridae. However, most of the reported fossil geometrids are questionable. Evers (1907) assigned a specimen from Zanzibar Island to the extant genus *Hyperythra* and regarded this specimen as *H. lutea*, but Kozlov (1988) identified it as *Geometridites* sp. In addition, *Phalaenites proserpinae* Heer, 1861 was also considered as *Geometridites* sp. by Kozlov (1988). Lacking strong evidence, Sohn et al. (2012) disputed the Geometridae affiliation of *Problongos baudiliensis* Mérit & Mérit, 2008. Kusnezov (1941) treated *Angerona electrina* Giebel, 1862 as Macrolepidoptera *incertae sedis*. Grimaldi and Engel (2005) mentioned three specimens of Geometridae from Early Miocene Dominican amber (15–20 Mya), and provided pictures of these specimens.

**Table 1.** Fossil records of Geometridae.

	Subfamily	Genus	Species	Life cycle	Epoch	Locality/Country	Reference	Note
1	Ennominae	* Angerona *	† *A. electrina*	adult	possibly Holocene	not stated	Giebel 1862; Sohn et al. 2012	Kusnezov (1941) treated this species as Macrolepidoptera *incertae sedis*.
2	Ennominae	† *Eogeometer*	† *E. vadens*	larva	Late Eocene-Early Oligocene	Baltic	Fischer et al. 2019	
3	Ennominae	* Hyperythra *	*H. lutea* ?	adult	Late Pleistocene	Tanzania	Evers 1907	Kozlov (1988) considered this specimen as *Geometridites* sp.
4	Ennominae	† *Problongos*	† *P. baudiliensis*	adult	Late Miocene	France	Mérit and Mérit 2008	Sohn et al. (2012) disputed the Geometridae affiliation of *Problongos baudiliensis*.
5	Larentiinae	*Hydriomena* ?	† H. ? protrita	adult	Late Eocene	USA	Cockerell 1922	
6	unassigned	† *Geometridites*	† *G. jordani*	adult	Late Pliocene	Germany	Kernbach 1967	
7	unassigned		† *G. repens*	larva	Late Pliocene	Germany	Kernbach 1967	
8	unassigned		† *G. larentiiformis*	adult	Late Eocene	United Kingdom	Jarzembowski 1980	
9	unassigned	† *Phalaenites*	† *P. crenatus*	adult	Early Miocene	Croatia	Heer 1849	
10	unassigned		† *P. obsoletus*	adult	Early Miocene	Croatia	Heer 1849	
11	unassigned		† *P. proserpinae*	adult	Late Oligocene-Early Miocene	France	Heer 1861	Kozlov (1988) considered this species as *Geometridites* sp.
12	unassigned	not stated	not stated	adult	Miocene	Dominican Republic	Grimaldi and Engel 2005: 568, fig. 13: 24	
13	unassigned	not stated	not stated	larva	Miocene	Dominican Republic	Grimaldi and Engel 2005: 588, fig. 13: 58	
14	unassigned	not stated	not stated	adult	Miocene	Dominican Republic	Grimaldi and Engel 2005: 588, fig. 13: 59, 60	
15	unassigned	not stated	not stated	adult	Late Cretaceous	New Zealand	Harris and Raine 2002: 461, fig. 1	Sohn et al. (2012) treated this as a questionable geometrid fossil.
16	unassigned	not stated	not stated	pupa	Late Pleistocene	Japan	FIRGNE 1990: 101, fig. 10.3.1	FIRGNE is Fossil Insect Research Group for Nojiri-ko Excavation.
17	unassigned	not stated	not stated	not stated	Pleistocene-Holocene	Benin and Guinea	Handlirsch 1908: 1133	
18	unassigned	not stated	not stated	not stated	Middle Eocene	Lutetian	Lewis 1992: 16	

†: extinct.

Here we describe a new genus and species of Geometridae based on an adult specimen preserved in Dominican amber. The age of Dominican amber-bearing deposits is the late Early Miocene through early Middle Miocene, ca 15 to 20 Mya (Iturralde-Vinent and Macphee 1996). Dominican amber, with exquisite preservation, contains a very rich Miocene biota with more than 400 described insect species (Arillo and Ortuño 2005). To date, 30 fossil records within seven superfamilies of Lepidoptera have been reported in Dominican amber (Poinar et al. 1991; Poinar and Brown 1993; Hall et al. 2004; Grimaldi and Engel 2005; Peñalver and Grimaldi 2006; Sohn et al. 2012). All these fossil records belong to the lepidopteran clade Ditrysia.

## Materials and methods

The type specimen in amber described herein is housed in Laboratorio Dominicano De Ambar Y Gemas, Santo Domingo, Dominican Republic. The specimen was examined and photographed by using a Nikon SMZ 18 dissecting microscope with an attached Nikon DS-Ri2 digital camera system and a Leica M205A with an attached Leica DMC5400 digital camera system. These devices used cool white LED illuminators. Cool white transmitted light passed through the specimen from the bottom up, and cool white light, emitted from double optical fibers, irradiated the specimen from two sides simultaneously. Images were prepared for illustration using Adobe Photoshop CS6. Wing index is defined as the ratio of wing width/wing length. The body length was measured from the apex of head to the terminal end of abdomen. Family-level classification follows van Nieukerken et al. (2011). Wing venation nomenclature is based on Wootton (1979).

## Systematic paleontology

### Order Lepidoptera Linnaeus, 1758

**Suborder Glossata Fabricius, 1775**

**Infraorder Heteroneura Tillyard, 1918**

**Superfamily Geometroidea Leach, 1815**

**Family Geometridae Leach, 1815**

**Subfamily Ennominae Duponchel, 1845**

#### Genus Miogeometrida

Taxon classificationAnimaliaLepidopteraGeometridae

Zhang, Shih & Shih gen. nov.

BEA6E9E7-96FB-5CD3-A300-BA08ADFF6729

http://zoobank.org/9AB3E411-9767-4CFF-88F9-6E37C92081D1

##### Type species.

*Miogeometrida chunjenshihi* Zhang, Shih & Shih, sp. nov.

##### Etymology.

The generic name is a combination of the prefix “Mio-” in reference to the Miocene, and “geometrid” in reference to the family name. The gender is masculine.

##### Diagnosis of genus.

Body length ca 5.7 mm, wingspan ca 20 mm. Antenna filiform. Forewing without fovea, R_1_ not anastomosing with Sc, no areole formed by veins R_1_ and Rs, R_1_ and Rs_1_ completely coincident, M_2_ arising midway between M_1_ and M_3_, anal veins 1A and 2A fused for its entire length. Hind wing with Rs running close to Sc + R_1_, and M_2_ absent.

##### Remarks.

The new genus can be distinguished from most extant or extinct geometrids by the absence of an areole formed by veins R_1_ and Rs. As *Miogeometrida* gen. nov. lacks M_2_ on the hind wing, affiliation with other subfamilies than Ennominae is excluded. *Miogeometrida* gen. nov. differs from most genera of Ennominae in its forewing without fovea and R_1_ not anastomosing with Sc. *Miogeometrida* gen. nov. is similar to genera such as *Ekboarmia* (Ennominae, Boarmiini, covered in Skou et al. 2017) and *Iridopsis* (Ennominae, Boarmiini, covered in McGuffin 1977) in venation and the absence of a fovea, but the antennae of the latter are pectinated in males. Apart from this, extant *Iridopsis* are much larger than *Miogeometrida* gen. nov. on average. *Miogeometrida* gen. nov. also shows similarities with genera such as *Milocera*, *Chelotephrina*, *Tephrina*, *Isturgia* and *Macaria* (Ennominae, Macariini, covered in Krüger 2001) in the forewing with R_1_ and Rs_1_ completely coincident and hind wing with two anal veins, but *Miogeometrida* gen. nov. differs from them in its forewing with 1A and 2A fused for their entire lengths.

Grimaldi and Engel (2005) mentioned three specimens of Geometridae from Dominican amber and provided a photo and a line drawing of one specimen (Grimaldi and Engel 2005: 588, fig. 13: 59, 60). According to the line drawing (Grimaldi and Engel 2005: 588, fig. 13: 60), the stem of M is present on its forewing. But in *Miogeometrida* gen. nov., the loss of the stem of M results in the formation of one large discal cell. *Miogeometrida* gen. nov. differs from the Eocene species *Geometridites larentiiformis* by the absence of the areole and R_1_ completely coincident with Rs_1_ on the forewing. Mérit and Mérit (2008) reported Miocene *Problongos*, whose forewing length is twice as long as that of *Miogeometrida* gen. nov. (22 mm vs. 8.9 mm).

#### Miogeometrida chunjenshihi

Taxon classificationAnimaliaLepidopteraGeometridae

Zhang, Shih & Shih sp. nov.

B712CC67-87E9-575A-AF28-5C0848A1CE9E

http://zoobank.org/B0B59F0C-43DB-4B48-8031-8EED7747EB43

Figures 1 , 2

##### Material.

***Holotype***: LEP-DA-2019001, male. Mouthparts, mid- and hind legs, abdominal sternum missing.

##### Etymology.

The specific name is dedicated to Chun Jen Shih, father of YuHong Shih, for his discovery of the type specimen and his efforts and dedication in collecting and promoting Dominican amber, especially his classification system for Dominican blue amber with the best quality known as Sky Blue Amber.

##### Locality and horizon.

La Búcara mine, Cordilliera Septentrional, Dominican Republic. La Toca Formation; late Early Miocene to early Middle Miocene.

##### Diagnosis.

As for the genus (see above), by monotypy.

##### Description.

Body slender, length 5.7 mm; wingspan ca 20 mm. Forewing length 8.9 mm; hind wing length 6.2 mm.

Head densely scaled; antenna filiform, partly preserved; compound eyes oval; chaetosemata unidentifiable; ocelli absent.

**Figure 1.**
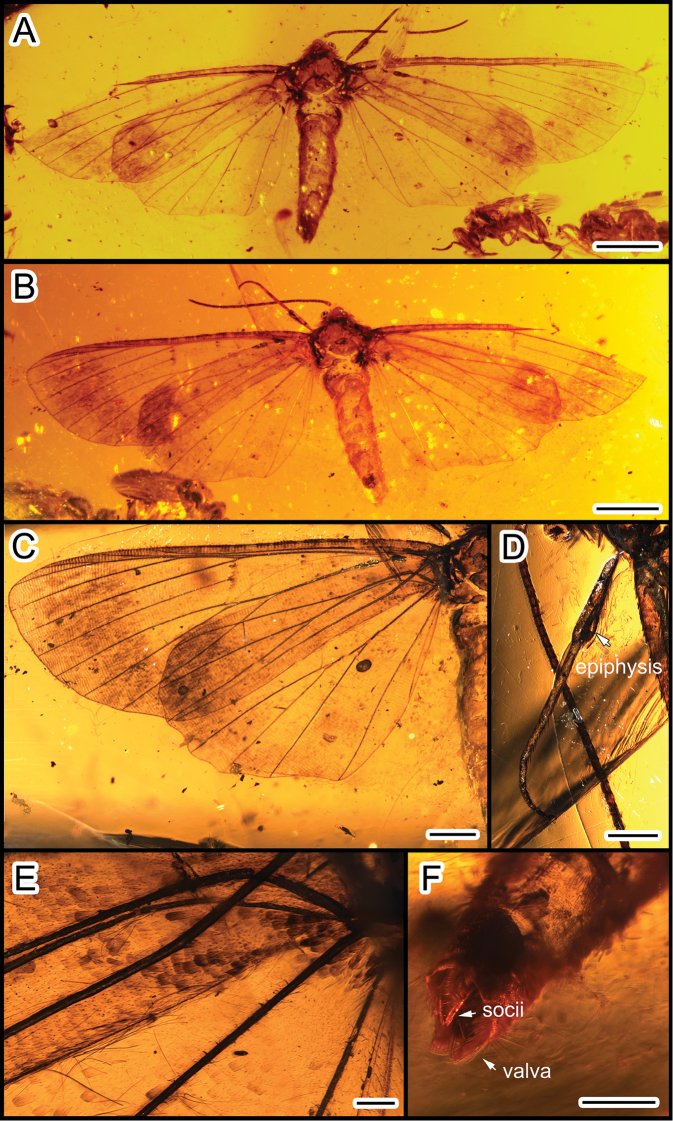
*Miogeometrida chunjenshihi* gen. et sp. nov., holotype, LEP-DA-2019001 **A** dorsal view **B** ventral view **C** forewing **D** foreleg **E** basal part of fore- and hind wings with scales **F** male genitalia, dorsal view. Scale bars: 2 mm (**A, B**); 1 mm (**C**); 0.5 mm (**D, F**); 0.2 mm (**E**).

Mesoscutum large, with median suture. Mesoscutellum rhomboid, smaller than mesoscutum. Metascutum triangular. A comb-like epiphysis with setae on its inner side, arising from the inner wall of the foretibia (Fig. 1D); tarsus with five tarsomeres, pretarsus with a pair of claws and a median arolium.

**Figure 2.**
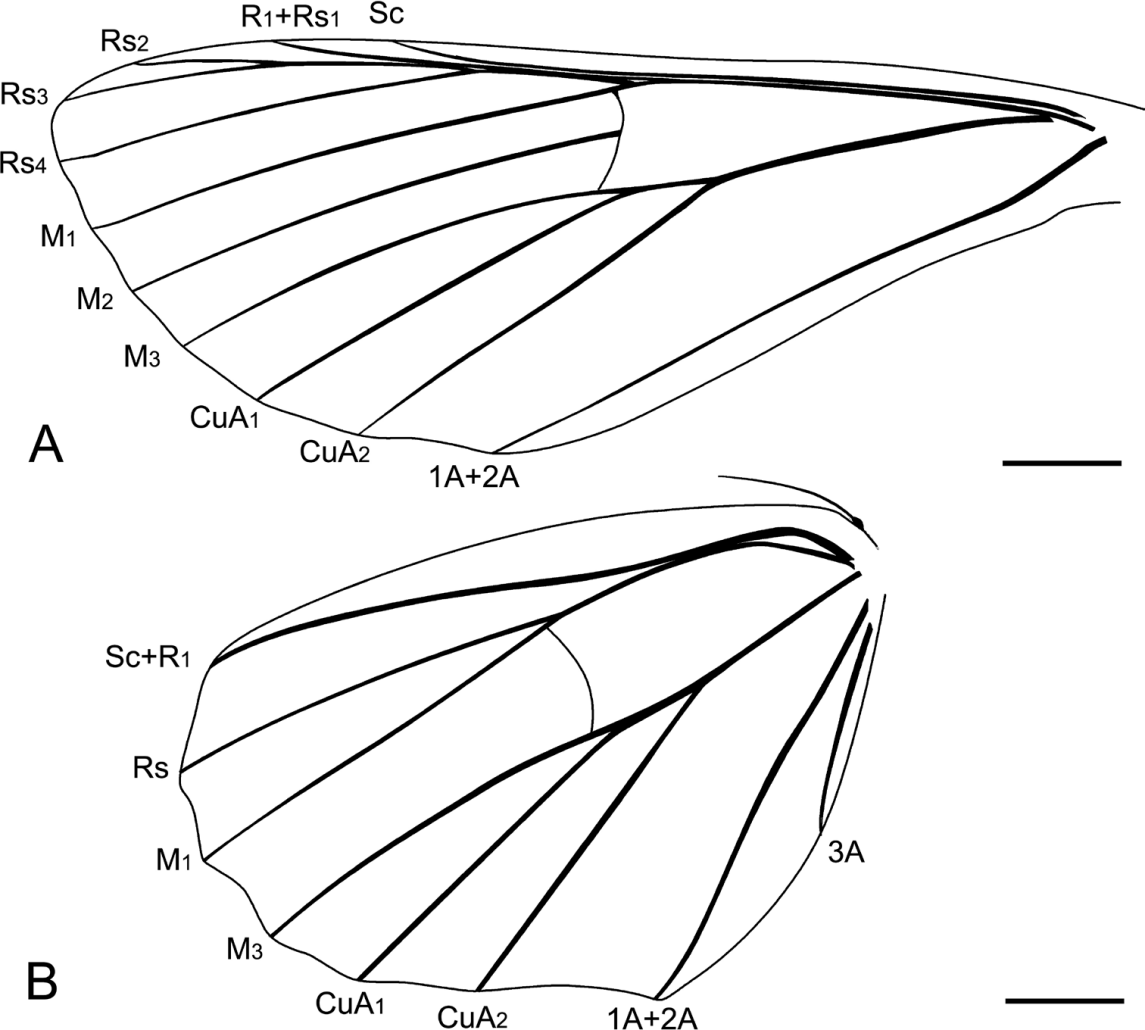
*Miogeometrida chunjenshihi* gen. et sp. nov., line drawings of LEP-DA-2019001 **A** forewing **B** hind wing. Scale bars: 1 mm.

Scales covering both fore- and hind wings, hair-like scales visible on the base of wings (Fig. 1C, E). Forewing elongate-triangular with the termen slightly sinuous; forewing index 0.37; fovea absent. Forewing with eleven veins (Figs 1C, 2A); discal cell approximately half as long as forewing; Sc not anastomosing with R_1_; no areole formed by R_1_ and Rs; R_1_ and Rs_1_ completely coincident; Rs_2_ and Rs_3_ with common stem; M 3-branched; M_1_ continuous with stem of R; M_2_ arising midway between M_1_ and M_3_; CuA bifurcating, CuA_1_ originating near the end of discal cell, CuA_2_ originating beyond the middle of discal cell; CuP absent; 1A and 2A fused for their entire lengths. Hind wing broad (Figs 1C, 2B), with outer margin concave between veins, apical angle rounded; hind wing index 0.66; Sc+R_1_ strongly bent at its base; Rs approximated to Sc+R_1_ at the base; M_2_ absent; M_1_ and M_3_ almost parallel; CuA_1_ and CuA_2_ as in forewing; anal veins 1A+2A and 3A present. Wing coupling present, one strong frenular bristle on the anterior margin of the hind wing, retinaculum of the forewing indistinct.

Male genitalia (Fig. 1F) with valva simple; uncus reduced; socii long, slender, with bristles on the inner side.

## Discussion

*Miogeometrida* gen. nov. can be assigned to Geometroidea based on forewing without spinarea (i.e., forewing-metathoracic aculeate locking device) and hind wing with basal part of the upper edge of discal cell markedly convex upwards, which are autapomorphies of the Geometroidea (Rajaei et al. 2015). In Geometroidea, the abdominal tympanal organ is an important diagnostic character, but the lateral and ventral parts of the abdomen of our specimen of *Miogeometrida* gen. nov. are damaged. It is thus impossible to determine whether a tympanal organ is present or not.

Although the essential apomorphy of Geometridae, i.e. a unique tympanal organ at the base of the abdomen, is not preserved for characterization, we chose to assign *Miogeometrida* gen. nov. to Geometridae. Based on the preserved and observable characters, *Miogeometrida* gen. nov. shows many similarities with Geometridae: (1) The size of *Miogeometrida* gen. nov. is in the common range of geometrids (wingspan ranges in most species from 20 to 45 mm; Heppner 2008a); (2) Hind wings of *Miogeometrida* gen. nov. are rounded as is the case in most species of Geometridae (Heppner 2008a); (3) *Miogeometrida* gen. nov. matches the major characters of geometrids in venation, such as forewing Rs_4_ stalked with Rs_2_ and Rs_3_, M_2_ not arising nearer to M_3_ than M_1_, and hind wing Sc bent strongly at its base (Minet and Scoble 1999). Although the first two similarities are also true for many other Lepidoptera, they can separate *Miogeometrida* gen. nov. from most sematurids and uraniids of Geometroidea.

We provide additional evidence to exclude three related Geometroidea families, i.e., Sematuridae, Uraniidae and Epicopeiidae. Sematuridae is a small family comprising only six extant genera and 40 species (van Nieukerken et al. 2011). An autapomorphy of Sematuridae are distally thickened antennae with swollen scape and elongate first flagellomere (Minet and Scoble 1999) – *Miogeometrida* gen. nov. does not have such an antenna. In addition, *Miogeometrida* gen. nov. with a wingspan of ca 20 mm, is obviously far smaller than sematurids whose wingspan range from 42 to 100 mm (Heppner 2008b). Moreover, *Miogeometrida* gen. nov. does not possess tails on the hind wings as found in most sematurids. In Uraniidae, the base of Rs_4_ is connate or stalked with M_1_, but separate from the other branches of Rs on the forewing, an apomorphy of the group (Minet and Scoble 1999). In *Miogeometrida* gen. nov., however, Rs_4_ is stalked with Rs_2+3_ on the forewing, which does not conform with the state in Uraniidae. Similarly, *Miogeometrida* gen. nov. can be distinguished from Epicopeiidae whose Rs_4_ is never stalked with Rs_1_ + Rs_2_ + Rs_3_.

Ennominae is the largest subfamily of Geometridae, comprising ca 10,000 species worldwide, classified in approximately 1100 genera (Pitkin 2002). *Miogeometrida* gen. nov. shows many similarities with some extant taxa. We assign *Miogeometrida* gen. nov. to Ennominae based on the absence of M_2_ on its hind wing that is considered as the traditionally diagnostic feature for this subfamily (Holloway 1994, Pitkin 2002). However, we cannot assign the new genus to tribe, mostly due to the poor preservation of its detailed morphological characters.

## Supplementary Material

XML Treatment for Miogeometrida

XML Treatment for Miogeometrida chunjenshihi
